# Phytotoxicity of Essential Oils on Selected Weeds: Potential Hazard on Food Crops

**DOI:** 10.3390/plants7040079

**Published:** 2018-09-22

**Authors:** María Dolores Ibáñez, María Amparo Blázquez

**Affiliations:** Departament de Farmacologia, Facultat de Farmàcia, Universitat de València, Avd. Vicent Andrés Estellés s/n, 46100 Burjassot, Valencia, Spain; mijai@alumni.uv.es

**Keywords:** winter savory, peppermint, essential oils, food crops, weed control, phytotoxicity

## Abstract

The chemical composition of winter savory, peppermint, and anise essential oils, and in vitro and in vivo phytotoxic activity against weeds (*Portulaca oleracea*, *Lolium multiflorum*, and *Echinochloa crus-galli*) and food crops (maize, rice, and tomato), have been studied. Sixty-four compounds accounting for between 97.67–99.66% of the total essential oils were identified by Gas Chromatography-Mass Spectrometry analysis. Winter savory with carvacrol (43.34%) and thymol (23.20%) as the main compounds produced a total inhibitory effect against the seed germination of tested weed. Menthol (48.23%), menthone (23.33%), and *iso*-menthone (16.33%) from peppermint only showed total seed germination inhibition on *L. multiflorum*, whereas no significant effects were observed with *trans*-anethole (99.46%) from anise at all concentrations (0.125–1 µL/mL). Low doses of peppermint essential oil could be used as a sustainable alternative to synthetic agrochemicals to control *L. multiflorum.* The results corroborate that in vivo assays with a commercial emulsifiable concentrate need higher doses of the essential oils to reproduce previous in vitro trials. The higher in vivo phytotoxicity of winter savory essential oil constitutes an eco-friendly and less pernicious alternative to weed control. It is possible to achieve a greater in vivo phytotoxicity if less active essential oil like peppermint is included with other active excipients.

## 1. Introduction

The potential hazard to the environment and human health, as well as the emergence of resistant weeds, are still the main problems of the overuse of synthetic herbicides used to improve global crop productivity. The continuous use of glyphosate, marketed in 1974 as a highly effective broad-spectrum herbicide [1], has made particular populations, such as the annual ryegrass (*Lolium rigidum* L.) in Australia [2] or barnyardgrass (*Echinochloa crus-galli* (L.) Beauv.) in cotton fields of the midsouthern United States [3], become glyphosate-resistant [4]. Recently, glyphosate resistance has been described in many world-wide species, like common ragweed (*Ambrosia artemisiifolia* L.) in several row crops of the south-eastern USA, following other still unknown mechanisms of action [5]. Together, resistant problems; potential health risks including skin irritancy, muscle atrophy, and nerve axons damage with prolonged exposure; and the use of a surfactant to enhance penetration [6] can even induce acute poisoning in humans, as well as chronic and sub-chronic toxicity, which has been reported in mammals after the consumption of contaminated food [7]. As these problems have appeared, some other weed killers have been synthesised as alternative solutions. However, the latest generation of the synthetic triketone herbicide family (sulcotrione, mesotrione, and tembotrione) also has negative impacts in microbial edaphic communities or plants, and even their degradation products can be more toxic than those of the parent, leading to similar environmental problems to glyphosate [8].

Resistance to other agrochemicals has also been reported in *Lolium* spp., exhibiting resistance to ALS-inhibiting herbicides that hinder acetolactate synthase (ALS), the enzyme common to the biosynthesis of the branch-chain amino acids (valine, leucine, and isoleucine) and ACCase (acetyl-coenzyme A carboxylase) inhibitors [9]. Similarly, common purslane (*Portulaca oleracea* L.) has developed resistance against linuron, a selective pre- and early post-emergent herbicide, in carrot (*Daucus carota*) fields [10]. In summary, weeds have evolved resistance in most of the known herbicide sites of action, being reported in 75 crops of 69 countries [11]. So, it is necessary to find more eco-friendly and less hazardous natural alternatives than synthetic herbicides, without promoting the emergence of resistance. In terms of natural compounds, essential oils are well-known for their multiple biological properties: anti-inflammatory, anticancer, antiviral, repellent, antibacterial, antifungal, or antioxidant, and have been widely used in the perfumery, cosmetics, pharmaceutical, and food industry, also being investigated to control crop pests [12]. In this sense, *Satureja montana* L. (Lamiaceae) was the highest effective larvicide of the essential oils tested against *Culex quinquefasciatus* [13] and was particularly active against several of the most damaging phytopathogenic fungi (*Fusarium*, *Alternaria*, *Rhizoctonia*, *Phytophthora*, and *Botrytis* spp.) that are able to destruct plant tissues, mainly cereals [14].

According to their phytotoxic capacity, *Satureja* spp. have potential as natural herbicides due to their main components, carvacrol and thymol, which are able to decrease in vitro germination and the growth of lambsquarters, common purslane, and barnyardgrass [15].

Another interesting bioresource is peppermint (*Mentha piperita* L.) essential oil, because it is able to exert a higher antimicrobial effect against *Escherichia coli*, *Staphylococcus aureus*, and *Candida albicans*, as well as upper antioxidant activity in DPPH free radical scavenging and β-carotene/linoleic acid systems, compared to other plant species, such as *Myrtus communis* [16]. These characteristics make peppermint essential oil a possible suitable bio-preservative to prevent post-harvest food decay. In this sense, it could be used to delay mold formation and reduce the incidence of infections when included as part of a coating as a previous experiment with only low amounts of the volatile that were enough to control fungal rot affecting *Vitis labrusca* L. maintained fruit quality during storage [17]. Regarding our topic, it is one of the most phytotoxic essential oils of 12 aromatic species, including *Thymus vulgaris* and *Salvia officinalis* against *Amaranthus retroflexus*, *Avena fatua*, *Bromus secalinus*, and *Centaurea cyanus* [18]. In fact, a dose-dependent inhibition of seed germination percentage, root and shoot lengths, and dry weight of field bindweed (*Convolvulus arvensis* L.), purslane, and jungle rice (*E. colonum* L.) has been observed at different concentrations (0, 300, 600, 900, 1200, 1500, and 1800 µL/L) of peppermint essential oil, whereas horticultural crops such as tomato (*Lycopersicon esculentum* Mill.) and radish (*Raphanus sativus* L.) were even more susceptible [19].

On the other hand, it is also interesting to expand the research with anise (*Pimpinella anisum* L.) essential oil since it is an annual medicinal plant belonging to the Apiaceae family and popularly known for its widespread use in the food and drink industry [20]. Its essential oil has shown higher antioxidant activity in in vitro models than the synthetic antioxidants butylated hydroxyanisole (BHA) and butylated hydroxytoluene (BHT), being possibly used for protecting fat-containing foods [21]. Antibacterial effects [22], as well as antifungal capacity, against *Saccharomyces cerevisiae*, *Aspergillus niger* [23], *Bipolaris/Dreschslera sorociniana*, *Fusarium subglutinans*, *Fusarium vertricilioides*, *Fusarium oxysporum*, *Fusarium tricinctum*, *Fusarium sporotrichioides*, *Fusarium equiseti*, *Fusarium incarnatum*, *Fusarium proliferatum*, and *Macrophomina phaseolina* have been also demonstrated [24], especially against *Saccharomyces cerevisiae*, which was effectively inhibited by anethole, the main component of aniseed, with an MFC value of 200 µg/mL [25]. In addition, it has been able to exert an insecticidal effect against young larvae of the Colorado potato beetle [26]. According to its phytotoxic activity, it has been described as one of the least active Mediterranean essential oils: it has shown a lower in vitro inhibitory effect in the seed germination of garden cress (*Lepidium sativum*), even promoting its germination and/or radicle elongation, as well as in food crops, such as lettuce (*Latuca sativa*) [27]. Despite its low phytotoxic potential, it has exhibited an effective competitive ability on common purslane, common lambsquarters, black nightshade, and barnyardgrass, being more suitable in low-input agricultural systems [28]. However, due to the harmful capacity of herbicides to remain inactivated for months in the soil and food products later consumed, anise essential oil has been recently included as one of the volatiles able to decompose and/or inhibit the function of the herbicide, together with ginger, peppermint, juniper, and lemongrass essential oils, through the dissolution and alteration of its chemical structure. In this case, the primary degradation product after the application of these essential oils is aminomethylphosphonic acid (AMPA), which is detected in much lower amounts in soil than glyphosate [29].

So, the aims of this work are firstly to test the in vitro phytotoxic activity (of previously analysed commercial essential oils, winter savory, peppermint, and anise, in order to assure their main compounds by GC/MS) against seed germination and seedling growth of *P. oleracea*, a cosmopolitan annual weed of tropical and subtropical climates; *L. multiflorum*, because *Lolium* spp. has been ranked as one of the specimens most frequently exhibiting herbicide resistance in many countries [30,31,32]; and *E. crus-galli*, a serious weed of irrigation crops, especially rice. Secondly, we aim to corroborate in vivo the previous in vitro phytotoxic effect using a commercial emulsifiable concentrate with the more phytotoxic essential oils and finally, we will study their potential hazard against maize (*Zea mays* L.), rice (*Oryza sativa* L.), and tomato (*Solanum lycopersicum* L.) seeds in order to obtain selective bioherbicides for food crops.

## 2. Results

### 2.1. Chemical Composition of Winter Savory, Peppermint, and Anise Essential Oils

Sixty-four compounds accounting for 97.67–99.66% of the total commercial winter savory, peppermint, and anise essential oils were identified by GC/MS analysis. Components are clustered (Table 1) in homologous series of monoterpene hydrocarbons, oxygenated monoterpenes, sesquiterpene hydrocarbons, oxygenated sesquiterpenes, diterpene hydrocarbons, aromatic compounds, and others, and are listed according to Kovat’s retention index calculated in GC on an apolar HP-5MS column.

In winter savory essential oil, the monoterpene compounds (95.06%), both oxygenated (71.90 ± 0.08%), with 16 compounds identified and hydrocarbons (23.16 ± 0.33%) including 13 components, were the main qualitative and quantitative fractions found. The phenolic compounds carvacrol (43.34 ± 0.09%) and thymol (23.20 ± 0.06%), followed by their biogenetic precursors *p*-cymene (11.41 ± 0.01%) and γ-terpinene (5.78 ± 0.01%), were the main compounds of winter savory essential oil. Together, the oxygenated monoterpenes, linalool, became the next major constituent of this fraction, although at a far lower percentage (2.34 ± 0.01%). Other compounds were detected in lower quantities, such as terpinen-4-ol (1.04 ± 0.01%) and *cis*-sabinene hydrate (0.20 ± 0.01%). Among the sesquiterpene fraction (3.46%), only relatively large amounts of the sesquiterpene hydrocarbon β-caryophyllene (2.81 ± 0.01%) were found, while the rest ranged from 0.03% for γ-cadinene to 0.31% for the oxygenated sesquiterpene caryophyllene oxide. Abietatriene (0.06%) and eugenol (0.05%) were the only diterpene hydrocarbon and phenylpropanoid detected in winter savory essential oil, respectively.

Regarding peppermint essential oil, oxygenated monoterpenes (94.77%) with 12 compounds identified were the main qualitative and quantitative phytochemical group found. Sesquiterpene hydrocarbons at a far lower percentage (2.49%) constituted the next phytochemical group. Oxygenated sesquiterpenes and others were found at percentages lower than 1% (0.26 and 0.15%, respectively). Finally, neither monoterpene hydrocarbons nor aromatic compounds were detected in the commercial peppermint essential oil analysed here. Between the 24 identified compounds in *M. piperita*, the oxygenated monoterpene menthol (48.23 ± 0.36%), followed by menthone (23.33 ± 0.59%) and its diasteromer *iso*-menthone (16.33 ± 0.03%), were the main compounds. Among the sesquiterpene hydrocarbons, only β-caryophyllene (1.26 ± 0.04%) reached a percentage higher than 1%. E-nerolidol, spathulenol, and caryophyllene oxide were the only oxygenated sesquiterpenes identified, with each one reaching 0.09%.

Finally, in anise essential oil, the main phytochemical group was by far the aromatic fraction (99.57 ± 0.05%), with four compounds identified, in which the leading component was *trans*-anethole (99.46 ± 0.05%). The rest contained within this fraction did not reach percentages higher than 0.1%: methyl chavicol (0.04%), *p*-anis aldehyde (0.04%), and *cis*-anethole (0.03%). Only two more compounds, the sesquiterpene hydrocarbons, α-*cis*-bergamotene and α-*trans*-bergamotene, were identified in *P. anisum* essential oil.

### 2.2. Seed Germination and Seedling Growth Inhibition of P. oleracea, L. multiflorum, and E. crus-galli, and Maize, Rice, and Tomato with Essential Oils

The phytotoxic effect of winter savory, peppermint, and anise essential oils was evaluated in vitro against three known harmful herbs: *P. oleracea*, *L. multiflorum*, and *E. crus-galli*. In this set of trials, the high phytotoxicity of winter savory essential oil (Table 2) was highly remarkable, exhibiting a total inhibitory effect against the seed germination of the tested weeds at all doses (0.125, 0.25, 0.50, and 1 µL/mL) assayed.

Furthermore, it was also noteworthy that the complete inhibition of the seed germination of *L. multiflorum* by peppermint essential oil was exhibited at all doses (0.125, 0.25, 0.50, and 1 µL/mL) applied (Table 2). Besides, significant differences between the control and the highest dose (1 µL/mL) of peppermint essential oil tested were found in the seed germination of both *P. oleracea* and *E. crus-galli*, although no significant effect at lower doses on the seed germination of *P. oleracea* and *E. crus-galli* was observed (Table 2).

**Figure 1:**
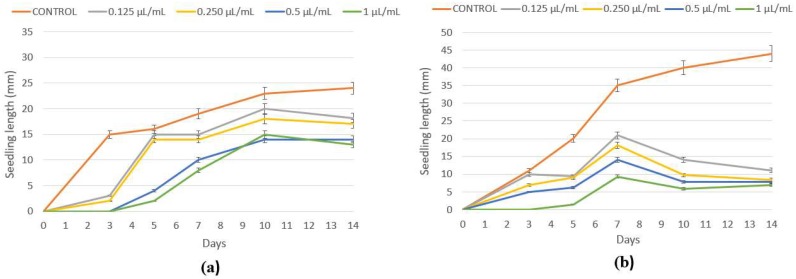
(**a**) *P. oleracea* and (**b**) *E. crus-galli* seedling growth with peppermint essential oil. Control and treated with peppermint essential oil at 0.125, 0.25, 0.50, and 1 µL/mL.

In addition, a stronger phytotoxic effect was found with peppermint essential oil against the seedling growth (hypocotyl and radicle) of *P. oleracea* and *E. crus-galli*, so it could be employed as a potential post-harvest treatment. According to *P. oleracea* seedling growth, significant differences were found between the control and the higher doses (0.50 and 1 µL/mL) tested (Table 3, Figure 1). Lower doses of peppermint essential oil (0.125 and 0.25 µL/mL) assayed did not cause a significant reduction in hypocotyl growth of *P. oleracea* seeds (37.25%), whereas a moderate (50.98%) inhibitory effect was observed when the highest dose (1 µL/mL) was applied. A similar result was found in radicle elongation, with a percentage inhibition of 43.48% at the two higher concentrations (Table 3). *E. crus-galli* seedling growth was more sensible to peppermint essential oil, experiencing a significant reduction in both hypocotyl and radicle development with respect to the control (Table 3, Figure 1b). There was no major difference in either hypocotyl or radicle enlargement when comparing concentrations (0.125, 0.25, 0.50, and 1 µL/mL), achieving between 75.40–86.64% and 71.13–82.10% inhibition of hypocotyl and radicle expansion, respectively.

**Figure 2:**
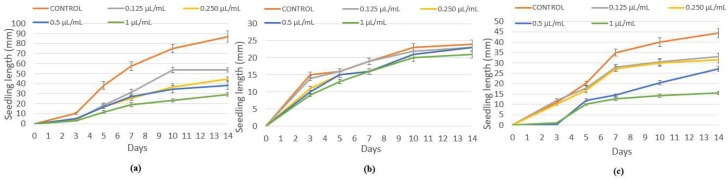
(**a**) *L. multiflorum*, (**b**) *P. oleracea*, and (**c**) *E. crus-galli* seedling growth. Control and treated with anise essential oil at 0.125, 0.25, 0.50, and 1 µL/mL.

Anise essential oil showed the absence of a significant herbicidal effect for both the doses and weed species tried (Table 2). It displayed no significant phytotoxic activity against seed germination (Table 2) of weeds affecting food crops; however, the seedling growth (hypocotyl and radicle) of *L. multiflorum* was significantly inhibited in a dose-dependent manner (Table 4), as well as the hypocotyl development of *E. crus-galli* that was depleted (63.23%) at the highest (1 µL/mL) dose and the radicle elongation at all concentrations applied with a percentage inhibition between 36.29 to 65.40% (Table 4, Figure 2c).

The previous selective inhibitory effect at the concentrations applied displayed by peppermint essential oil against the three assayed weeds with a total *L. multiflorum* seed germination inhibition, was not observed against the three selected crops (Table 5). Tomato was the most sensible crop, with an almost complete seed germination inhibition (96.84%) at the highest dose assayed (1 µL/mL), followed by maize (79.31%) and rice (36.96%). Peppermint essential oil significantly affected the growth of both the hypocotyl and radicle of rice and tomato (Table 5). The maize radicle was also significantly disturbed by these treatments in comparison to the control, whereas the volatile oil did not affect the hypocotyl growth of maize. It is interesting to note the results of peppermint essential oil against rice and *L. multiflorum*, which show a lower phytotoxic effect in rice, with a seed germination inhibition percentage between 18.48% to 16.30% at the doses of 0.125, 0.25, and 0.50 µL/mL (Table 5); concentrations that cause a total seed germination inhibition of *L. multiflorum*, one of the principal weeds that affect this crop (Table 2).

### 2.3. Seed Germination and Seedling Growth Inhibition of P. oleracea, L. multiflorum, and E. crus-galli, and Maize, Rice, and Tomato with an Emulsifiable Concentrate Including Winter Savory or Peppermint Essential Oils

The phytotoxic effect exhibited by winter savory and peppermint essential oils in in vitro trials were corroborated by in vivo conditions using two different emulsifiable concentrates elaborated by SEIPASA, a pioneer Spanish Company in the development, manufacturing, and marketing of environmentally friendly agro-inputs in order to produce healthy food.

The results of the emulsifiable concentrate containing a final dose of 5 or 10 µL/mL of essential oil were compared with a control watered with water and a blank without the corresponding essential oils.

The emulsifiable concentrate of winter savory succeeded in inhibiting the seed germination of *P. oleracea* within 33 days at both concentrations, corroborating the previous in vitro results in which there was also total inhibition of the weed germination of common purslane (Table 2).

**Figure 3:**
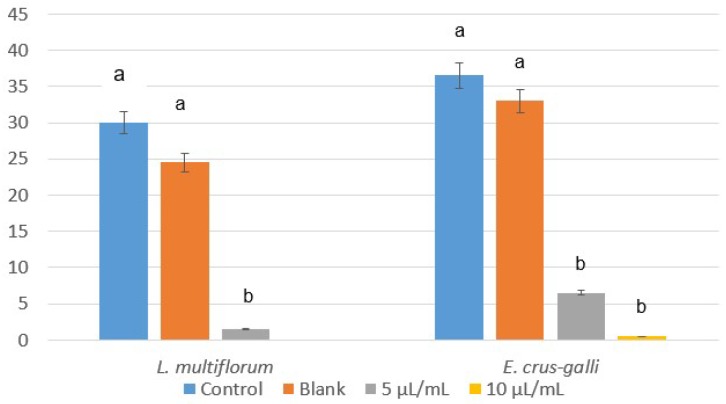
Values of seed germination (%) of *L. multiflorum* and *E. crus-galli* control and blank, and treated with the emulsifiable concentrate of winter savory essential oil at 5 and 10 µL/mL. Means followed by different letters in each column indicate that they are significantly different at *p* < 0.05 according to T3 Dunnet and Tukey tests.

**Figure 4:**
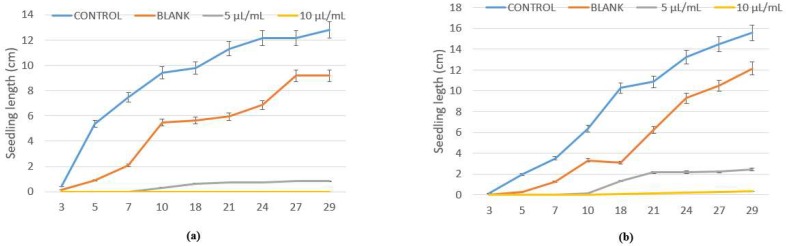
Hypocotyl growth (cm) of (**a**) *L. multiflorum* and (**b**) *E. crus-galli* control and blank, and treated with winter savory essential oil at 5 and 10 µL/mL.

Despite *L. multiflorum* and *E. crus-galli* being more tolerant species in in vivo conditions, the emulsifiable concentrate of winter savory also significantly inhibited their seed germination, with percentages of 95–100% and 82–99% at 5 and 10 µL/mL, and without a significant effect with the blank (Figure 3). Similar results were found for hypocotyl growth (Figure 4), making the formulate with winter savory essential oil an ecological alternative to synthetic herbicides, which have already demonstrated a detrimental influence on the environment, crops, and human health.

Regarding peppermint essential oil, due to the less phytotoxical effect, it was emulsified with other herbicidal compounds (blank use by SEIPASA Company) and the formulate was able to inhibit the seed germination of the three weeds in a dose-dependent manner, with percentages between 77–100% for *P. oleracea*, 90–95% against *E. crus-galli*, and total inhibition over *L. multiflorum*, corroborating the in vitro results in which peppermint essential oil was more active against *L. multiflorum* than *P. oleracea* and *E. crus-galli*.

**Figure 5:**
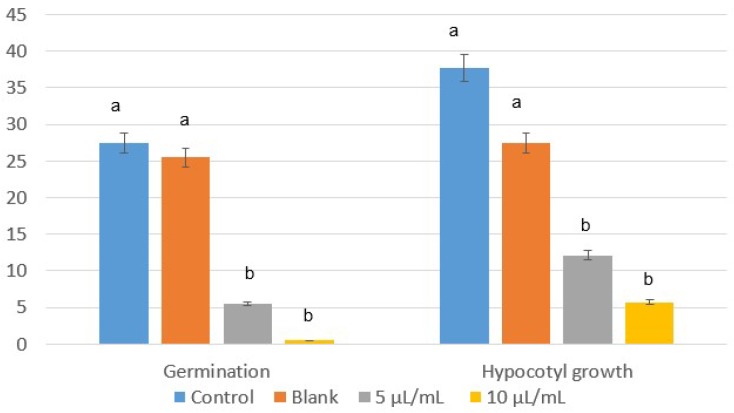
In vivo effect of the emulsifiable concentrate of winter savory essential oil over the germination and hypocotyl growth of tomato seeds. Values are mean of ten replications ± error deviation after 30 days of incubation. Means followed by different letters in the same column indicate that they are significantly different at *p* < 0.05 according to T3 Dunnet and Tukey tests.

According to the herbicidal effect of the emulsifiable concentrate of winter savory, a new set of trials was carried out with this formulate and three food crops. Unfortunately, a total seed germination inhibition was obtained with maize and rice, and between 80 and 98% of the tomato was inhibited at 5 and 10 µL/mL, respectively, with no significant effect with the blank (Figure 5).

## 3. Discussion

The phenolic compounds carvacrol (43.34 ± 0.09%) and thymol (23.20 ± 0.06%) were the main compounds identified in the winter savory essential oil analysed here. It is well-known that the strong spicy flavour of winter savory is determined by the prevailing carvacrol/thymol chemotype [33], which is variable in relation to the stage of development of the plant, harvesting time, and field environment conditions, including circumstances such as a variation in altitude: in fact, a higher content of linalool and other compounds found here in lower quantities (Table 1), such as terpinen-4-ol (1.04 ± 0.01%) and sabinene hydrate (0.20 ± 0.01%), has been detected in higher amounts in *S. montana* essential oil at a higher altitude, while both the major ones identified here, carvacrol and thymol, were quantified in lower percentages [34].

Apart from that, these phenolic compounds found in winter savory essential oil are considered the main bioactive monoterpenes that provide *S. montana* with a wide range of pharmacological and biological properties, such as natural antimicrobial activity [35] against gram-positive (*Staphylococcus aureus* and *Bacillus cereus*) and gram-negative bacteria (*Salmonella infantis* and *Escherichia coli* O157:H7) [36] that is useful in the treatment of foodborne diseases, as well as anti-inflammatory activity for certain transcription factors [37]. Furthermore, recently, some new thymol and carvacrol derivatives, including the carbamate moiety, have been synthesized with stronger inhibitory effects on acetylcholinesterase [38]. Related to their phytotoxicity, both carvacrol and thymol have shown total suppression of the seed germination and seedling growth of *Amaranthus retroflexus*, *Chenopodium album*, and *Rumex crispus* [39], coinciding with our authors [27] and also with previous [40] studies, in which oregano essential oil with 60.42% of carvacrol exhibited a total inhibition of *P. oleracea*, *L. multiflorum*, and *E. crus-galli* at the same in vitro doses assayed. These results indicate that winter savory is an effective broad-spectrum herbicide as it occurs with glyphosate that also exerts inhibitory effects on the seed germination of crops, such as wheat (*Triticum durum* L.), pea (*Pisum sativum* L.), lettuce (*Latuca sativa* L.) [41], and even trees, for instance *Pinus pinaster*, in which it is able to induce in vitro shoot chlorosis and drooping [42]. So, this formulate could be an alternative to glyphosate with less environmental and human health problems.

Several researches corroborate that the biological properties of peppermint essential oil are due to its chemical composition, especially its major components menthol, menthone, and *iso*-menthone [43,44], conferring the high percentage of menthol to peppermint essential oil immunostimulant effects in animals [45] and the herbicidal effect in Mediterranean weed, such as *Amaranthus retroflexus* L., *Solanum nigrum* L., and *P. oleracea* under controlled conditions [46]. The results obtained convert peppermint essential oil into a sustainable alternative that can solve the recent *L. multiflorum* resistance to glyphosate on rice paddy leaves [47]. Not only do our results match with other studies in reference to larger compounds, but also in reference to smaller ones, such as E-nerolidol, spathulenol, and caryophyllene oxide, which were the only oxygenated sesquiterpenes identified in peppermint essential oil [48,49].

Finally, although *trans*-anethole, the main compound in commercial anise essential oil (99.46 ± 0.05%) analysed here and in other previous works [50] has demonstrated strong antifungal activity through the inhibition of the mycelial growth of a wide range of fungi [51] and could be used as a preservative in food preparation and processing [49], our results together with other research [52] revealed no significant phytotoxic activity against seed germination of selected weed.

## 4. Materials and Methods

### 4.1. Essential Oil

Commercial samples of winter savory (*Satureja montana* L.) (Batch 0054366), peppermint (*Mentha piperita* L.) (Batch 0058567), and anise (*Pimpinella anisum* L.) (Batch 0059857) essential oils supplied by Guinama (Valencia, Spain) were stored at 4 °C until chemical analysis and phytotoxic assays.

With the purpose of decreasing volatility, winter savory and peppermint essential oils were included in an emulsifiable concentrate industrially prepared by Seipasa Company that was stable at room temperature and adequate for in vivo weed control assays.

### 4.2. Seeds

Mature seeds of annual weeds of *Portulaca oleracea* L., *Lolium multiflorum* Lam., and *Echinochloa crus-galli* (L.) Beauv., were purchased from Herbiseed (website: www.herbiseed.com).

Mature seeds of ‘Perseo-type’ maize (*Zea mays* L.) and ‘Albufera-type’ rice (*Oryza sativa* L.) were obtained from the cereals in Sueca (Valencia, Spain). ‘Huevo de toro-type’ tomato (*Solanum lycopersicum* L.) seeds were directly acquired from the fruit found in the inner part in Utiel (Valencia, Spain).

### 4.3. Gas Chromatography- Mass Spectrometry

GC-MS analysis was carried out with a 5973N Agilent apparatus, equipped with a capillary column (95 dimethylpolysiloxane- 5% diphenyl), Agilent HP-5MS UI (30 m long and 0.25 mm i.d. with 0.25 µm film thickness). The column temperature program was 60 °C for 5 min, with 3 °C/min increases to 180 °C, and then 20 °C/min increases to 280 °C, which was maintained for 10 min. The carrier gas was Helium at a flow-rate of 1 mL/min. Split mode injection (ratio 1:30) was employed. Mass spectra were taken over the *m/z* 30–500 range with an ionizing voltage of 70 eV.

### 4.4. Identification

The individual compounds were identified by MS and their identity was confirmed by a comparison with their Kovat’s retention index calculated using co-chromatographed standard hydrocarbons relative to C8–C32 *n*-alkanes, and mass spectra with reference samples or with data already available in the NIST 2005 mass spectral library and in the literature [53].

### 4.5. In Vitro Assays: P. oleracea, L. multiflorum, E. crus-galli, Maize, Rice, and Tomato Seed Germination and Seedling Growth with Essential Oils

Sets of 20 seeds (10 for maize), each with five replicates (ten replicate in maize) per treatment, were homogenously distributed in Petri dishes (9 cm diameter) between two layers of filter paper (Whatman No.1) moistened with 4 mL of distilled water and with 0 (control), 0.125, 0.250, 0.5, and 1 µL/mL of winter savory, peppermint, and anise essential oils. Petri dishes were sealed with parafilm and incubated in a germination chamber Equitec EGCS 301 3SHR model, according to previous assays [54], alternating between 30.0 ± 0.1 °C 16 h in light and 20.0 ± 0.1 °C 8 h in dark, with (*E. crus-galli*, maize, and rice) and without (*P.oleracea*, *L. multiflorum*, and tomato) humidity. To evaluate the herbicidal activity of the essential oils, the number of germinated seeds was counted and compared with those of untreated seedlings. Emergence of the radicle (≥1 mm) was used as an index of germination and seedling length (hypocotyl and/or radicle) data were recorded after 3, 5, 7, 10, and 14 days in each replicate.

### 4.6. In Vivo Assays: P. oleracea, L. multiflorum, E. crus-galli, Maize, Rice, and Tomato with an Emulsifiable Concentrate of Winter Savory or Peppermint

Ten seeds of each species (*P. oleracea*, *L. multiflorum*, *E. crus-galli*, maize, rice, and tomato) with ten replicates per treatment were randomly chosen and placed in pods (9 cm diameter) with 40 g of substrate. They were place less than 1 cm below Substrate Projar Professional containing coir and peat make, fertilizer N-P-K: 14 + 16 + 18 + micronutrients, and dolomitic limestone with a sorption capacity of 183 g/10 min. A set of 10 pods was watered on the first day with 20 mL of water (control), 20 mL commercial products (Nosbur OE 12 NS (32% *w*/*w*), Emulson AG/CAL/E (7% *w*/*w*), Emulson CO 36 (13% *w*/*w*) or Emulson AG/CAL/E (2.2% *w*/*w*), Alpicare 410H (21.7% *w*/*w*), Emulson AG/7720/A (2.6% *w*/*w*) respectively) without essential oils (blank), and 20 mL of emulsifiable concentrate with winter savory (48% *w*/*w*) or peppermint essential oil (73.5 % *w*/*w*) at 5 and 10 µL/mL. A tray was used every five pods to hold and separate them when watering. In order to prevent leaching, the pods were covered with plastic film. Over a period of 33 or 20 days (winter savory or peppermint), each tray was watered with 250 mL of water every two days. The greenhouse conditions were: 23.3 °C average indoor temperature, 18.1 °C minimum indoor temperature, 29.7 °C maximum indoor temperature, 57.2% average humidity, 80.9 µmol/m^2^/s PAR (Photo Active Radiation), and 135.6 W/m^2^ intensity of radiation.

To evaluate the herbicidal effect, the number of germinated seeds in 5 µL /mL and 10 µL /mL pod trays was counted and compared with those of control and blank samples. Emergence of the hypocotyl (≥1 mm) was used as an index of germination and seedling length data were recorded every two days, coinciding with watering days over 33 or 20 days.

### 4.7. Statistical Analysis

Experiments were conducted with five replicates and ten replicates in vitro and in vivo, respectively. Data were subjected to one-way analysis of variance (ANOVA) with SPSS statistics 22 software. Tukey’s post hoc test was used when variances remained homogeneous (Levene’s test) and T3 Dunnett’s post hoc one was employed if not, assuming equal variances. Differences were considered to be significant at *p* ≤ 0.05.

## 5. Conclusions

The results in vitro showed that winter savory and peppermint essential oils can be effective bioherbicides. Peppermint essential oil at lower doses could be used to control *L. multiflorum* in rice. The emulsifiable concentrate based on winter savory essential oil tested in in vivo assays corroborates that this effective broad-spectrum herbicide constitutes an eco-friendly and less pernicious alternative to glyphosate in weed control.

## Figures and Tables

**Table 1:** Chemical composition of commercial *S. montana*, *M. piperita*, and *P. anisum* essential oils.

RI	Compound	*S. montana* Peak Area (%)	*M. piperita* Peak Area (%)	*P. anisum* Peak Area (%)
Monoterpene hydrocarbons		23.16 ± 0.33	-	-
931	α-Thujene	0.88 ± 0.01	-	-
939	α-Pinene	0.77 ± 0.00	-	-
953	Camphene	0.32 ± 0.00	-	-
979	β-Pinene	0.11 ± 0.08	-	-
993	Myrcene	1.39 ± 0.01	-	-
1005	α-Phellandrene	0.21 ± 0.00	-	-
1012	δ-3-Carene	0.08 ± 0.00	-	-
1019	α-Terpinene	1.62 ± 0.01	-	-
1029	*p*-Cymene	11.41 ± 0.01	-	-
1033	Limonene	0.35 ± 0.26	-	-
1053	*trans*-Ocimene	0.04 ± 0.00	-	-
1063	γ-Terpinene	5.78 ± 0.01	-	-
1090	Terpinolene	0.21 ± 0.01	-	-
Oxygenated monoterpenes		71.90 ± 0.08	94.77 ± 0.07	-
1035	1,8-Cineole	0.07 ± 0.00	-	-
1070	*cis*-Sabinene hydrate	0.20± 0.01	-	-
1098	*trans*-Sabinene hydrate	0.07 ± 0.01	-	-
1101	Linalool	2.34 ± 0.01	-	-
1146	Camphor	0.02 ± 0.00	-	-
1150	Isopulegol	-	0.80 ± 0.02	-
1155	Menthone	-	23.33 ± 0.59	-
1164	*iso*-Menthone	-	16.33 ± 0.03	-
1169	Borneol	0.71 ± 0.02		-
1176	Menthol	-	48.23 ± 0.36	-
1179	Terpinen-4-ol	1.04 ± 0.01		-
1184	*iso*-Menthol	-	0.52 ± 0.03	-
1186	*p*-Cymen-8-ol	0.02 ± 0.01		-
1188	*neo*-*iso*-Menthol	-	0.22 ± 0.01	-
1191	α-Terpineol	0.41 ± 0.01	0.26 ± 0.01	-
1203	*trans*-Dihydrocarvone	0.03 ± 0.01	0.09 ± 0.01	-
1237	Methyl ether Thymol	0.26 ± 0.01	-	-
1242	Pulegone	-	0.85 ± 0.06	-
1246	Neral	0.06 ± 0.01	-	-
1249	Carvone	0.06 ± 0.01	-	-
1256	Piperitone	-	0.68 ± 0.06	-
1297	Thymol	23.20 ± 0.06	-	-
1298	Menthyl acetate	-	3.38 ± 0.26	-
1307	*iso*-Menthyl acetate	-	0.06 ± 0.07	-
1314	Carvacrol	43.34 ± 0.09	-	-
1374	Carvacryl acetate	0.08 ± 0.01	-	-
Sesquiterpene hydrocarbons		3.11 ± 0.02	2.49 ± 0.04	0.09 ± 0.00
1338	δ-Elemene	-	0.13 ± 0.01	-
1351	α-Cubebene	-	0.08 ± 0.02	-
1388	β-Bourbonene	-	0.34 ± 0.02	-
1390	β-Elemene	-	0.14 ± 0.01	-
1416	α-*cis*-Bergamotene	-	-	0.01 ± 0.00
1420	β-Caryophyllene	2.81 ± 0.01	1.26 ± 0.04	-
1437	α-*trans*-Bergamotene	-	-	0.08 ± 0.00
1454	α-Humulene	0.11 ± 0.01	-	-
1495	Viridiflorene	0.05 ± 0.01	-	-
1500	α-Muurolene	-	0.11 ± 0.00	-
1509	β-Bisabolene	0.06 ± 0.00	-	-
1514	γ-Cadinene	0.03 ± 0.01	0.12 ± 0.00	-
1524	δ-Cadinene	0.06 ± 0.00	0.30 ± 0.01	-
Oxygenated sesquiterpenes		0.35 ± 0.02	0.26 ± 0.01	-
1565	E-Nerolidol	-	0.09 ± 0.01	-
1578	Spathulenol	0.04±0.01	0.09±0.00	-
1583	Caryophyllene oxide	0.31±0.01	0.09±0.01	-
Diterpene hydrocarbons		0.06±0.01	-	-
2067	Abietatriene	0.06±0.01	-	-
Aromatic compounds		0.05±0.00	-	99.57 ± 0.05
1197	Methyl Chavicol	-	-	0.04 ± 0.00
1253	*p*-Anis aldehyde	-	-	0.04 ± 0.00
1255	*cis*-Anethole	-	-	0.03 ± 0.00
1286	*trans*-Anethole	-	-	99.46 ± 0.05
1359	Eugenol	0.05 ± 0.00	-	-
1406	Methyl Eugenol	-	-	-
Others		0.09 ± 0.01	0.15±0.02	-
980	1-Octen-3-ol	0.09 ± 0.01	-	-
1275	*n*-Decanol	-	0.15 ± 0.02	-
	Total	98.73 ± 0.40	97.67 ± 0.08	99.66 ± 0.05

RI, retention index relative to C8-C32 n-alkane on HP-5MS column; values are mean ± standard deviation of three samples.

**Table 2:** In vitro effects of peppermint, anise, and winter savory essential oils against *Portulaca oleracea*, *Lolium multiflorum*, and *Echinochloa crus-galli* seed germination.

Seed Germination (% ± e.d.)			
**Concentration (µL/mL)**	***Portulaca oleracea***		
	Winter Savory	Peppermint	Anise
Control	85.00 ± 2.74 a	85.00 ± 2.74 a	85.00 ± 2.74 a
0.125	0.00 ± 0.00 b	81.00 ± 2.45 a,b	82.00 ± 3.74 a
0.25	0.00 ± 0.00 b	80.00 ± 3.54 a,b	85.00 ± 5.24 a
0.5	0.00 ± 0.00 b	75.00 ± 3.87 a,b	82.00 ± 4.34 a
1	0.00 ± 0.00 b	70.00 ± 3.16 b	81.00 ± 1.87 a
**Concentration (µL/mL)**	***Lolium multiflorum***		
	Winter savory	Peppermint	Anise
Control	67.00 ± 5.15 a	67.00 ± 5.15 a	67.00 ± 5.15 a
0.125	0.00 ± 0.00 b	0.00 ± 0.00 b	65.00 ± 6.89 a
0.25	0.00 ± 0.00 b	0.00 ± 0.00 b	64.00 ± 4.30 a
0.5	0.00 ± 0.00 b	0.00 ± 0.00 b	62.00 ± 4.34 a
1	0.00 ± 0.00 b	0.00 ± 0.00 b	60.00 ± 3.54 a
**Concentration (µL/mL)**	***Echinochloa crus-galli***		
	Winter savory	Peppermint	Anise
Control	86.00 ± 3.32 a	86.00 ± 3.32 a	86.00 ± 3.32 a
0.125	0.00 ± 0.00 b	82.00 ± 3.74 a,b	89.00 ± 1.87 a
0.25	0.00 ± 0.00 b	82.00 ± 2.55 a,b	88.00 ± 1.23 a
0.5	0.00 ± 0.00 b	80.00 ± 1.58 a,b	83.00 ± 2.55 a
1	0.00 ± 0.00 b	72.00 ± 2.00 b	85.00 ± 4.47 a

Values are mean of five replications ± error deviation after 14 days of incubation. Means followed by different letters in the same column indicate that they are significantly different at *p* > 0.05 according to T3 Dunnet and Tukey tests.

**Table 3:** In vitro effects of peppermint essential oil against *P. oleracea* and *E. crus-galli* seedling growth.

	Seedling Growth (mm ± e.d.)	
**Concentration (µL/mL)**	**Peppermint**	
	***P. oleracea***	
	Hypocotyl	Radicle
Control	10.20 ± 0.58 a	13.80 ± 2.04 a
0.125	6.40 ± 0.25 a,b	11.80 ± 1.39 a,b
0.25	6.40 ± 0.25 a,b	10.20 ± 1.39 a,b
0.5	5.80 ± 0.20 b	7.80 ± 0.37 b
1	5.00 ± 0.00 c	7.80 ± 0.97 b
**Concentration (µL/mL)**	***E. crus-galli***	
	Hypocotyl	Radicle
Control	23.66 ± 3.80 a	20.78 ± 1.78 a
0.125	5.82 ± 0.71 b	6.00 ± 1.03 b
0.25	4.56 ± 0.37 b	3.86 ± 0.44 b
0.5	3.56 ± 0.72 b	4.34 ± 0.52 b
1	3.16 ± 0.69 b	3.72 ± 0.67 b

Values are mean of five replications ± error deviation after 14 days of incubation. Means followed by different letters in the same column indicate that they are significantly different at *p* > 0.05 according to T3 Dunnet and Tukey tests.

**Table 4:** In vitro effects of anise essential oil against *P. oleracea* and *E. crus-galli* seedling growth.

	Seedling Growth (mm ± e.d.)	
**Concentration (µL/mL)**	**Anise**	
	***P oleracea***	
	Hypocotyl	Radicle
Control	10.20 ± 0.58 a	13.80 ± 2.04 a
0.125	10.00 ± 0.89 a	13.40 ± 2.58 a
0.25	9.60 ± 0.68 a	13.60 ± 2.36 a
0.5	8.20 ± 0.37 a	14.60 ± 1.72 a
1	7.60 ± 1.60 a	13.40 ± 1.60 a
**Concentration (µL/mL)**	***L. multiflorum***	
	Hypocotyl	Radicle
Control	48.50 ± 3.35 a	39.14 ± 2.14 a
0.125	26.21 ± 0.94 b	27.65 ± 1.25 b
0.25	23.07 ± 1.17 b,c	21.29 ± 2.05 b,c
0.50	19.71 ± 2.45 c	18.72 ± 1.11 c
1	12.66 ± 0.61 d	16.66 ± 1.11 c
**Concentration (µL/mL)**	***E. crus-galli***	
	Hypocotyl	Radicle
Control	23.66 ± 3.80 a	20.78 ± 1.46 a
0.125	19.82 ± 0.95 a	13.24 ± 0.30 b
0.25	18.64 ± 1.17 a	12.90 ± 0.27 b
0.5	14.44 ± 0.30 a,b	12.70 ± 0.27 b
1	8.68 ± 2.24 b	7.19 ± 1.35 b

Values are mean of five replications ± error deviation after 14 days of incubation. Means followed by different letters in the same column indicate that they are significantly different at *p* > 0.05 according to T3 Dunnet and Tukey tests.

**Table 5:** In vitro seed germination and hypocotyl and radicle growth of maize, rice, and tomato seeds with peppermint essential oil.

Peppermint	Seed Germination (%) ± e.d.	Seedling Growth (mm ± e.d.)	
**Concentration (µL/mL)**	**Maize**		
	Germination	Hypocotyl	Radicle
Control	29.00 ± 4.20 a	5.10 ± 1.49 a	17.65 ± 3.24 a
0.125	15.00 ± 3.25 b	2.29 ± 0.83 a	4.85 ± 1.38 b
0.25	13.50 ± 3.08 b	2.31 ± 0.36 a	3.95 ± 0.89 b
0.5	7.50 ± 2.27 b	1.54 ± 0.74 a	2.06 ± 0.86 b
1	6.00 ± 2.21 b	1.72 ± 0.64 a	2.15 ± 0.74 b
**Concentration (µL/mL)**	**Rice**		
	Germination	Hypocotyl	Radicle
Control	92.00 ± 2.55 a	22.29 ± 5.72 a	33.52 ± 5.90 a
0.125	75.00 ± 3.16 b	5.64 ± 1.43 b	19.33 ± 2.30 b
0.25	75.00 ± 3.16 b	5.47 ± 1.74 b	16.48 ± 1.69 b
0.5	77.00 ± 6.44 b	6.85 ± 1.68 b	12.46 ± 1.75 b
1	58.00 ± 2.00 c	2.86 ± 0.23 b	7.25 ± 0.47 b
**Concentration (µL/mL)**	**Tomato**		
	Germination	Hypocotyl	Radicle
Control	95.00 ± 1.58 a	21.84 ± 2.00 a	33.14 ± 3.71 a
0.125	39.00 ± 12.59 b	4.30 ± 3.32 b	9.61 ± 5.23 b
0.25	31.00 ± 16.08 b	4.23 ± 1.68 b	7.70 ± 2.67 b
0.5	14.00 ± 4.30 c	1.48 ± 0.63 b	3.92 ± 1.34 b
1	3.00 ± 3.00 c	0.20 ± 0.20 b	1.12 ± 1.12 b

Values are mean of five replications ± error deviation after 14 days of incubation. Means followed by different letters in the same column indicate that they are significantly different at p < 0.05 according to T3 Dunnet and Tukey tests.
